# Potential mechanisms and targeting strategies of the gut microbiota in antitumor immunity and immunotherapy

**DOI:** 10.1002/iid3.1263

**Published:** 2024-07-19

**Authors:** Qian Yin, Jiao‐jiao Ni, Jie‐er Ying

**Affiliations:** ^1^ Postgraduate Training Base Alliance of Wenzhou Medical University (Zhejiang Cancer Hospital) Hangzhou Zhejiang China; ^2^ Department of Hepato‐Pancreato‐Biliary & Gastric Medical Oncology Zhejiang Cancer Hospital Hangzhou China

**Keywords:** antitumor immunity, cancer, cancer therapy, gut microbiota, immunotherapy

## Abstract

**Background:**

Immunotherapies, notably immune checkpoints inhibitors that target programmed death 1/programmed death ligand 1(PD‐1/PD‐L1) and cytotoxic T lymphocyte‐associated antigen 4 (CTLA‐4), had profoundly changed the way advanced and metastatic cancers are treated and dramatically improved overall and progression‐free survival.

**Aims:**

This review article aimed to explore the underlying molecular mechanisms by which the gut microbiota affects antitumor immunity and the efficacy of cancer immunotherapy.

**Methods:**

We summarized the latest knowledge supporting the associations among the gut microbiota, antitumor immunity, and immunotherapy. Moreover, we disscussed the therapeutic strategy for improving immunotherapy efficacy by modulating gut microbiota in cancer treatment.

**Results:**

The potential molecular mechanisms underlying these associations are explained in terms of four aspects: immunomodulation, molecular mimicry, mamps, and microbial metabolites.

**Conclusion:**

The gut microbiota significantly impacts antitumor immunity and alters the effectiveness of cancer immunotherapy.

## Graphical abstract

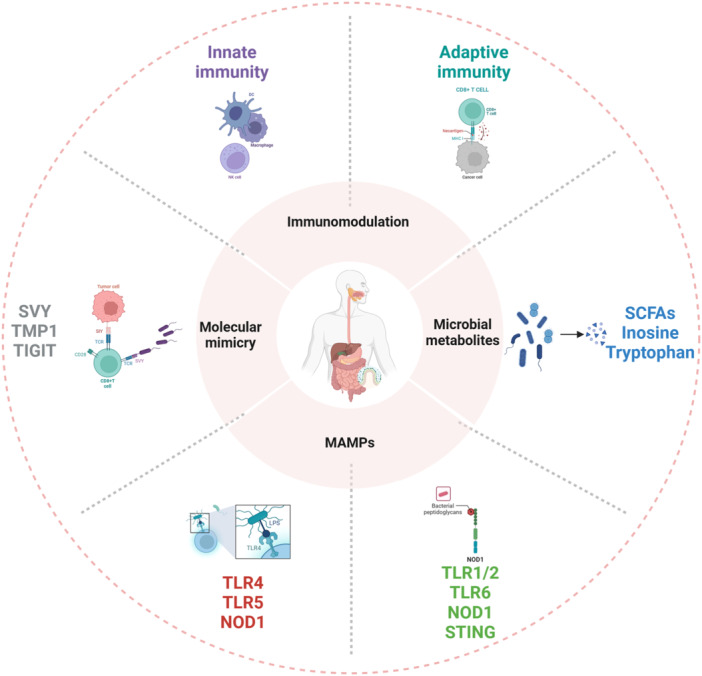

## 1 INTRODUCTION

**Figure 1:**
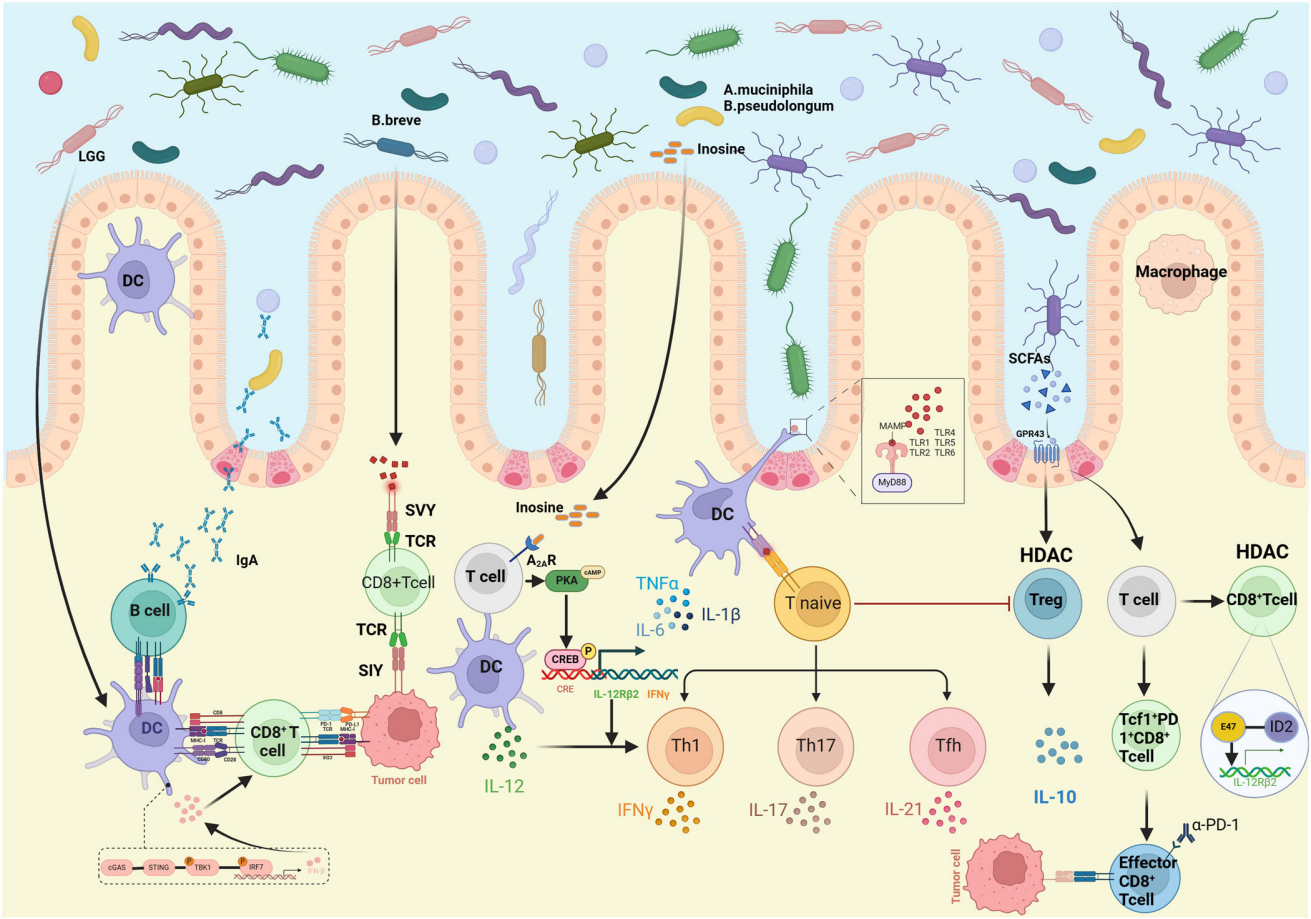
The molecular mechanisms of action of the gut microbiota in antitumor immunity and immunotherapy efficacy. The gut microbiota modulates antitumor immunity both by activating the innate immune system adaptive immune system, ultimately enhancing immunotherapy efficacy. Moreover, metabolites secreted by gut microbiota also regulate antitumor immune response, such as SCFAs and inosine. A2aR, adenosine 2A receptor; DC, dendritic cell; HDAC, histone deacetylases; IFNγ, Interferon‐γ; SCFAs, short‐chain fatty acids; TCR, T‐cell receptor; Th cell, helper T cell; Tfh cell, follicular helper T cell; TLRs, Toll‐like receptors; Treg cell, T regulatory cell; TNF‐α, tumor necrosis factor α.

In recent years, increasing attention has been given to the impact of the gut microbiota on host health and disease. The gut microbiota plays a critical role in regulating human physiology in various ways, and a multidimensional network of functions among the gut microbiota, the immune system, metabolism, and cancer has been identified.^1^, ^2^, ^3^ A large body of evidence indicates that the gut microbiota modulates the immune system, which has resulted in studies on the effect of cancer immunotherapy, especially immune checkpoint inhibitors (ICIs).^4^, ^5^, ^6^, ^7^, ^8^ The host immune system consists of both innate immunity and adaptive immunity components. The skin, gut, and lung linings, stomach acids, intestinal bacteria, and leukocytes (neutrophils) are involved in innate immunity that can detect and kill pathogens instantly. Immune protection that is “learned” is called adaptive immunity. Whenever the immune system is exposed to a disease, a type of bacterium, fungus, or virus for the first time, antibodies are produced so that the immune system responds faster and better the next time. ICIs release the immune brake response and inhibit tumor immune escape effectively by targeting lymphocyte activation gene‐3 (LAG3), cytotoxic T lymphocyte‐associated antigen‐4 (CTLA‐4), programmed cell death 1 (PD‐1)/programmed cell death ligand‐1 (PD‐L1), and other targets.^9^, ^10^, ^11^ Despite being well understood, the gut microbiota plays an important role in immunity,^12^ the underlying molecular mechanisms by which the gut microbiota affects antitumor immunity and the efficacy of cancer immunotherapy remain elusive. In our review, we summarize the latest knowledge supporting the associations among the gut microbiota, antitumor immune activity, and cancer immunotherapy and explain the potential mechanisms underlying this association (Figure 1).

## 2 THE GUT BARRIER AND GUT MICROBIOTA

The intestine is an innate barrier for maintaining environmental homeostasis in the gut and inhibiting pathogenic bacteria and toxins. A variety of barriers exist along the intestinal wall, including mechanical, chemical, microbial, and immune barriers. Disruption of the barrier results in leakage of intestinal contents, including the intestinal microbiota, metabolites, and immune cells, into the circulatory system, which may contribute to endotoxin translocation and systemic inflammation. Hence, immune regulation of the intestinal barrier and symbiotic microbiota is critical and has evolved conservatively.^13^

Over the past few years, it has been demonstrated that the gut microbiome plays a crucial role in the manipulation of the gut barrier and metabolic diseases via a variety of mechanisms.^14^ Leonardi et al.^15^ showed that mucosal fungi produce interleukin 22 (IL‐22) via CD4^+^ T helper. cells to strengthen the gut barrier in mice. Similarly, the production of short‐chain fatty acids (SCFAs) and antimicrobial peptides contributes to maintaining the integrity of the intestinal barrier.^14^ In contrast, outer membrane vesicles (OMVs) produced by pathogenic and symbiotic Gram‐negative bacteria may allow bacterial components to cross the intestinal barrier by disrupting the integrity of the mucosal epithelium and interacting with a variety of immune cells directly, thereby promoting pathological alterations.^16^ The gut is the primary site of interaction between the host and the external environment and houses more than 70% of the immune cells in the body.^17^ The gut microbiota interacts with the immune system in other parts of the body via the intestinal wall.^18^ The immune system is involved in maintaining the integrity of the intestinal barrier in a variety of ways. For example, a specific self‐sustaining macrophage that strictly localizes to blood vessels can form a tight anatomical barrier and may protect against bacterial translocation.^19^ The maturation of such perivascular macrophages in the intestinal mucosa is closely linked to the gut microbiota.^20^ Cytokines, which play a critical role in regulating the function of the intestinal barrier, have been well studied for more than two decades.^21^, ^22^ IL‐22, for example, regulates intestinal barrier function by inducing phospho‐Stat3 binding to the Il‐18 gene promoter and through a mechanism independent of IL‐18.^23^

The intensity and duration of the anticancer immune response to ICIs are highly dependent on the gut microbiota and on adequate baseline intestinal epithelium functions.^18^, ^24^ Notably, the effect of the intestinal barrier on the ICI response is less well understood, and interventions to adjust the intestinal wall to favor the ICI response may improve the prognosis of patients.^18^ Taken together, as the host's first line of defense against commensal and pathogenic bacteria, a good appreciation of the gut barrier will further our understanding of the role of the gut microbiota in antitumor immunity and in immunotherapy response.

## 3 THE GUT MICROBIOTA ACTIVATES THE INNATE IMMUNE SYSTEM TO MODULATE ANTITUMOR IMMUNITY

The innate immune system offers a key host immune response to the invasion of microbes in which microbe‐associated molecular patterns (MAMPs) are identified by host pattern recognition receptors (PRRs). Four well‐known PRRs are Toll‐like receptors (TLRs), nucleotide‐binding oligomerization domain (NOD)‐like receptors (NLRs), retinoic acid‐inducible gene I (RIG‐I)‐like receptors, and C‐type lectin receptors.^25^, ^26^ Among these PRRs, the main PRRs that recognize bacterial MAMPs are TLRs on the plasma membrane or endosome and NLRs in the cytoplasm.^25^

TLRs mainly localize to immune cells, including natural killer cells, macrophages, dendritic cells, mast cells, neutrophils, eosinophils, and basophils, and are distributed in intestinal epithelial cells.^14^, ^27^ Different TLRs mediate distinct responses to MAMPs derived from the gut microbiota. In the suppression of antitumor immunity, two classic examples are TLR4, which senses bacterial lipopolysaccharides (LPS), and TLR5, which recognizes bacterial flagellin. Mononuclear infiltrating cells differentiate into the immunosuppressive M2 phenotype to drive tumor progression through the binding of TLR4 to LPS.^28^, ^29^ Myeloid‐derived immunosuppressive cells (MDSCs) are a population of heterogeneous immature myeloid cells that have the capacity to suppress innate immunity via various mechanisms.^30^ LPS can bind to TLR4 on tumor cells to induce the accumulation of CXCR2^+^ PMN‐MDSCs to suppress the liver antitumor immune response^31^ and facilitate the recruitment of CD11b^+^Gr1^+^ MDSCs, thereby inhibiting the response to local antitumor T cells.^32^ The activation of TLR5 in leukocytes derived from bone marrow promoted carcinogenesis in a bacterial flagellin‐dependent manner in a chemically induced skin cancer model.^33^ In turn, the binding of MAMPs to TLRs can also promote antitumor immunity. The activation of TLR1/TLR2 by bacterial lipoprotein (BLP) can downregulate monocytic MDSCs (M‐MDSCs), which can help tumors escape immunosurveillance.^34^ *Lactobacillus* can promote the production of IL‐10 in a TLR6‐dependent manner to enhance the immune surveillance of cancerous lesions.^35^ It has been demonstrated that myeloid differentiation factor 88 protein (MyD88) plays a key role in the activation of TLR downstream signaling pathways.^14^ Oral supplementation with LPS can restore the IFN‐γ production ability of CD8^+^ T cells by increasing TLR4‐MyD88 signaling in infant mice.^36^

In addition, peptidoglycan (PGN) fragments derived from the gut microbiota recognize the intracellular receptor NOD1 to promote host innate immune activity.^37^ The initiation of arginase1‐dependent NOD1 can enhance immunosuppression driven by MDSCs, thereby promoting colorectal carcinogenesis.^38^ Interestingly, overexpression of NOD1 can also suppress the progression of estrogen‐dependent tumors. Mononuclear phagocytes (MPs), including monocytes (Mos), macrophages (Macs), and dendritic cells (DCs), play pivotal roles as innate immune cells in host homeostasis.^39^ STING agonists derived from microbiota and microbiota modulation with a high‐fiber diet can improve the efficacy of ICB in a Mo‐IFN‐γ‐NK‐DC‐dependent manner.^40^

## 4 THE GUT MICROBIOTA ACTIVATES THE ADAPTIVE IMMUNE SYSTEM TO MODULATE ANTITUMOR IMMUNITY

The intestine‐associated lymphoid tissue (GALT), including the lamina propria and Peyer's patch (PP), is the largest lymphoid organ in the body. GALT bridges the gap between innate and adaptive immunity through the recognition of PRRs, which trigger the functional maturation of dendritic cells (DCs) and initiate naive B cells and T cells.^41^ IgA‐producing plasma cells and effector T cells (or T regulatory (Treg) cells), which differentiate from naive B and T cells, can move from the outflow lymphatics of the GALT to mesenteric lymph nodes (MLNs) before eventually entering the peripheral blood through the thoracic duct and thereby participating in systemic immunity.^42^

B cells are key substances in adaptive humoral immunity and maintain intestinal homeostasis mainly through the production of large amounts of secretory immunoglobulin A (slgA) antibodies, which can coat the gut microbiota and soluble antigens.^43^ SIgA is a major component of the host mucosal defense system and helps defend the host against pathogens by limiting the growth of commensal bacteria and preventing them from penetrating the mucosal barrier.

Signaling changes from the intestinal luminal environment can cause T cells to differentiate into different types and thus initiate adaptive cellular immunity. Dysbiosis of the intestinal microbiota can promote primitive CD4^+^ T cells to differentiate into Treg cells or effector T cells. In GF mice, the lack of IL‐17^+^CD4^+^ T (Th17) cells coincided with an increase in FoxP3^+^ Treg cells, resulting in an imbalance in Th17/Treg homeostasis and blunted antitumor immunosurveillance during carcinogenesis.^44^, ^45^ Th17 cells are a particular type of CD4^+^ T‐cell that is engaged in the immune elimination of extracellular bacteria and fungi.^46^, ^47^ The upregulation of serum amyloid A (SAA) and reactive oxygen species (ROS) induced by segmented filamentous bacteria (SFB) can enhance the number of primitive CD4^+^ T cells that differentiate into Th17 cells through enterocyte‐SFB adhesion mechanisms.^48^ In contrast, FoxP3^+^ Tregs are significantly responsible for suppressing excessive immune responses. Polysaccharide A (PSA), which is synthesized by *B. fragilis* and Clostridium and is an important immune regulator, can promote the polarization of naive CD4^+^ T cells to FoxP3^+^ Tregs via exposure to DCs or TLR pathways.^49^ Dysregulation of the gut microbiota disrupts the interaction between mucosal addressin cell adhesion molecule 1 (MAdCAM‐1) on high endothelial venules and integrin α4β7 expressed by Treg17 cells and leads to the relocation of Treg 17 cells into tumors and tumor‐draining lymph nodes(tdLNs), thereby affecting ICI efficacy.^50^ CD8^+^ T cells are a key factor in antiviral and antitumor immunity for the differentiation of infected or tumor cells from normal cells.^51^ Reduced thymic maturation of CD8^+^ T cells can be reversed by bacterial peptidoglycan via the NOD signaling pathway in GF mice.^52^

The gut microbiota, host, and mucosal barrier are altered significantly under pathobiological conditions. Dendritic cells (DCs) present tumor antigens through MHC‐I and/or MHC‐II to CD8^+^ T cells and CD4^+^ T cells, triggering an antitumor response. The gut microbiota induces the release of immunomodulatory cytokines from intestinal epithelial cells or immune cells to alter the threshold for activation of immune cell subpopulations, thereby regulating the antitumor immune response. It has been demonstrated that *Bifidobacterium* can stimulate DC maturation and improve the cross‐presentation ability of DCs. Administration of *Bifidobacterium* and anti‐CD47 can facilitate IFNγ production in CD8^+^ T cells via the STING signaling pathway.^53^ Specifically, *Bifidobacterium bifidum* (*B. bifidum*) administration decreased the levels of IL‐10 and TNF‐α in tumor cells. In addition, the combination of anti‐PD‐1 and *B. bifidum* increased the levels of cytokine‐producing IFN‐γ^+^CD8^+^ and IL‐2^+^CD4^+^ tumor‐infiltrating T cells in a peptidoglycan‐dependent manner.^54^ Moreover, *Bacteroides fragilis* can boost the efficacy of CTLA‐4 blockade by affecting IL‐12‐dependent Th1 immune responses.^55^

A typical characteristic of T cell receptors (TCRs) is high cross‐reactivity. When bacterial antigens have high similarity with antigens expressed in tumors and undergo “molecular mimicry”, cross‐reactive T cells are elicited to recognize and kill tumor cells. The B16.SIY tumor neoantigen, which resembles the SVY epitope expressed in *Bifidobacterium breve*, can be recognized by cross‐reactive T cells and suppress the growth of tumors.^56^ The tail length tape measure protein 1 (TMP1) of a prophage in *Enterococcus hirae*, which has MHC I binding epitopes, has high homology with the proteasome subunit beta type‐4 (PSMB4)‐derived peptide. These compounds trigger CD8^+^ T‐cell antitumor activity and enhance the effectiveness of anti‐PD‐1 treatment.^57^ The FAP2 protein of *Fusobacterium nucleatum* can bind to the immune cell suppressor receptor TIGIT and protect tumor cells from NK cell‐mediated killing and T‐cell attack, promoting colorectal cancer development.^58^

## 5 MICROBIAL METABOLITES REGULATE THE ANTITUMOR IMMUNITY AND IMMUNOTHERAPY EFFICACY

Another typical mechanism by which the gut microbiota regulates the antitumor immunity is through the generation of metabolites. These microbial metabolites can interact with receptors on host cells, which can inhibit or activate signaling pathways, with both benefits and drawbacks for the health of the host.

When dietary fiber is fermented in the colon, it produces short‐chain fatty acids (SCFAs), which include acetate, propionate, and butyrate. It has been extensively demonstrated that SCFAs play a key role in maintaining intestinal mucosal integrity and immune homeostasis.^59^, ^60^, ^61^ The main mechanisms by which SCFAs modulate immune homeostasis include G protein‐coupled receptors (GPRs), histone deacetylases (HDACs), mammalian target of rapamycin (mTOR), and metabolic regulation.^62^ SCFAs can regulate Treg cell activity and upregulate the production of ILC3‐derived IL‐22 through the AKT and STAT3 signaling pathways in a GPR43‐dependent manner.^63^, ^64^, ^65^ Microbial fermentation‐derived SCFAs can also trigger GPR43‐dependent, antitumor responses by increasing the production of IFN‐γ^+^ CD8^+^ T cells and stem‐like T‐cell factor‐1^+^ PD‐1^+^ CD8^+^ T cells.^66^ In addition to GPR43, SCFAs can also bind to GPR109A to regulate the differentiation of immune cells and cytokine secretion.^67^ Butyrate, which is the most extensively documented SCFA, significantly promotes the antitumor immune activity of CD8^+^ T cells in an HDAC‐ID2‐dependent fashion by boosting the IL‐12 signaling pathway.^4^ Glycolytic metabolism plays a key role in regulating the expression of IFN‐r and effective T‐cell function.^68^, ^69^, ^70^ SCFAs can modulate the function and metabolism of immune cells via the mTOR signaling pathway. They can promote effective TH1 cell, TH17 cell, and IL‐10^+^ T‐cell differentiation through the inhibition of HDAC and the mTOR‐S6K pathway.^71^ Pentanoate and butyrate can suppress class I HDAC activity and enhance mTOR activity in CD8^+^ T cells, resulting in increased expression of effector molecules and the enhanced antitumor activity of CD8^+^ T cells.^72^

In addition to SCFAs, bile acids (BAs), particularly secondary BAs (SBAs), which are derived from BAs generated in the liver, play a crucial role in tumorigenesis and immunomodulation.^73^, ^74^, ^75^, ^76^, ^77^ Lithocholic acid (LCA) and deoxycholic acid (DCA) produced by SBAs have been discovered in *C. hiranonis*, *C. hylemonae*, and *C. scindens*.^78^ 3‐Oxolithocholic acid derived from LCA can bind to RORgt to inhibit Th17 cell differentiation, while LCA‐derived isoallolithocholic acid enhances Treg cell differentiation and FOXP3 expression by generating mitochondrial reactive oxygen species.^79^, ^80^ Another SBA, 3b‐hydroxydeoxycholic acid derived from DCA, promotes the production of RORγt Treg cells in a CNS‐1‐dependent manner through the suppression of farnesoid X receptor (FXR) activity in DCs.^77^ Notably, an increase in the number of FOXP3+ Treg cells can lead to the inhibition of antitumor immunity and decrease the efficacy of ICIs.^81^ The role of SBAs in tumor development is twofold. The mechanism by which SBAs promote tumorigenesis involves the NF‐kB, Wnt/β‐catenin, and intrinsic apoptotic pathways.^82^, ^83^, ^84^ On the other hand, the activation of FXR and TGR5 can inhibit tumorigenesis.^85^, ^86^ FXR activation can curtail CRC development through suppression of the Wnt/β ‐catenin signaling pathway and suppressor of cytokine signaling 3 (SOCS3) gene transactivation.^85^

Inosine, which is a purine metabolite of *B. pseudolongum* and *A. muciniphila*, enhances the tumor‐killing activity of T cells and the efficacy of ICI.^87^ Increased ectopic inosine caused by impaired function of the intestinal barrier binds to the adenosine 2A receptor (A2AR) expressed on T cells, resulting in the activation of the T‐cell antitumor response and enhanced efficacy of anti‐CTLA‐4 therapy. Interestingly, the action of inosine on T cells for potent antitumor immunity requires adequate CpG and DC costimulation, the involvement of IL‐12 receptors in Th1 differentiation, and the production of IFN‐γ.^87^ Another study also demonstrated that inosine offers an alternative carbon source for CD8^+^ T‐cell function in a glucose‐limited environment to support effective T‐cell growth and function as well as enhance the efficacy of ICIs.^88^ Moreover, inosine can also enhance tumor immunogenicity and ICI efficacy through tumor cell sensitization to cytotoxic T cells in a UBA6‐dependent manner.^88^

The aryl hydrocarbon receptor (AhR) is an environmental sensor of microbiota‐derived tryptophan metabolites and transcription factors. Recent studies have demonstrated that AhR is critical for regulating the development and function of immunity through ligands derived from the diet, microbiome, and metabolism of the host cell.^89^, ^90^, ^91^ AhR functions as a regulator of cell fate decisions to promote the polarization of resident memory T cells as well as inhibit T central memory cell differentiation, suggesting that AhR signaling pathway activation may have therapeutic implications in cancer immunotherapy.^92^ The gut microbiota can metabolize tryptophan to kynurenine, serotonin, or indole, all of which are AhR ligands.^93^ Recently, Hezaveh et al.^94^ revealed the impact of AhR on tumor‐associated macrophage (TAM) function in pancreatic ductal adenocarcinoma (PDAC). Inhibition of AhR can reduce tumor growth in PDAC and improve the response to ICB therapy. Indole produced by lactobacilli obviously promotes PDAC growth in mice, with an increase in the population of MDSCs and a reduction in the abundance of TNF‐α^+^IFNγ^+^CD8^+^ T cells.^94^ Indole‐3‐aldehyde(I3A) derived from *Lactobacillus reuteri* (Lr) can facilitate antitumor immune activity and improve the efficacy of ICIs in preclinical melanoma.^95^ Mechanistically, I3A secreted by Lr directly promotes the production of IFN‐γ in a CREB‐dependent fashion via CD8^+^ T‐cell‐specific AhR signaling.^95^

## 6 THE POTENTIAL MECHANISM BY WHICH THE GUT MICROBIOTA INFLUENCES THE IMMUNOTHERAPY EFFICACY

For decades, immunotherapy, which redirects the immune response to influence the lasting control of tumors, has been the most important advance in cancer treatment. ICIs prolong antitumor responses to immune systems by targeting negative modulator proteins.^9^ A series of pioneering studies have demonstrated that intestinal bacteria play an important role in modulating the efficacy of ICIs in various cancers^55^, ^96^, ^97^, ^98^, ^99^ and that treatment with ICI‐friendly bacteria may help reverse primary resistance to ICIs.^97^

PD‐1 and PD‐L1 are immune checkpoint proteins associated with the inhibition of the immune system and the transmission of suppressive signals to T cells.^100^ *Akkermansia muciniphila (A. muciniphila)* is one of the “star” enterobacteria of the intestinal flora and may exert a variety of health benefits, including the modulation of host immunity, but its molecular mechanisms remain to be understood. In GF mice, oral administration of *A. muciniphila* and *Enterococcus hirae (E. hirae)* enhances the efficacy of anti‐PD‐1 therapy in metastatic melanoma, NSCLC, and RCC. The potential mechanism by which *A. muciniphila* and *E. hirae* influence anti‐PD‐1 efficacy may be associated with the aggregation of central memory T cells and stimulation of IL‐12 secretion in mesenteric lymph nodes, lymph nodes in tumor drainage areas, and tumor beds of mice receiving transplants of ineffective patient colonies.^97^ Another study revealed that *A. muciniphila* induces anti‐IgG1 and antigen‐specific T‐cell responses in mice.^101^ However, this study did not reveal the molecular mechanism underlying the action of *A. muciniphila* on T cells. Recently, a study revealed the molecular mechanism by which *A. muciniphila* regulates host immunity. a15:0‐i15:0 PE, a diacyl phosphatidylethanolamine with two branched chains expressed on the *A. muciniphila* cell membrane, causes the release of specific inflammatory cytokines by acting on the nonclassic TLR2‐TLR1 heterodimer and, at low doses, “blunts” the activation threshold of immune cells.^102^

CTLA‐4 is expressed constitutively in Treg cells as an immune checkpoint that weakens the immune response.^103^ In addition to *A. muciniphila*, other specific bacteria, such as *B. fragilis* and *Bifidobacterium*, can augment the efficacy of ICIs in a mouse model. Immunogenic *Bacteroides thetaiotaomicron* or *B. fragilis* affects the Th1 cell antitumor immune response in an IL‐12‐dependent manner and enhance the efficacy of anti‐CTLA4 therapy.^55^ Oral supplementation with *Bifidobacterium* activated DCs and facilitated the antitumor function of tumor‐specific CD8^+^ T cells, thereby promoting anti‐PD‐L1 efficacy.^96^ The main toxic effect of ICI therapy is that it often leads to severe autoimmunity, most frequently colitis.^104^ The search for new strategies and specific mechanisms that can both mitigate adverse events of immunotherapy and improve clinical outcomes has been a longstanding endeavor. The microbiota plays a key role in mitigating overstimulation of the immune system by inducing immune tolerance through enhanced induction of Tregs at mucosal barrier sites and generation of immunomodulatory metabolites that enter the circulation.^12^, ^105^ A recent study shows that CTLA‐4 blockade‐induced colitis in mice is dependent on the composition of their intestinal microbiota. They further revealed that the mechanisms driving intestinal inflammation are activated IFNγ‐producing CD4^+^ T cells and a decrease in peripheral Tregs due to Fcγ receptor signaling. Anti‐CTLA4 nanobodies lacking the Fc domain can facilitate the response to antitumor without eliciting colitis.^106^ *Bifidobacterium* boosted the inhibitory activity of Treg cells in an IL‐10‐dependent manner to minimize anti‐CTLA4 treatment‐induced immunopathology while not compromising CTLA‐4 antitumor function in mice.^107^, ^108^ Polysaccharides on the cell surface of *B. bifidum* can also alleviate colitis through the induction of regulatory DC‐dependent Foxp3^+^ Treg cells.^87^

*L. rhamnosus GG (LGG)* is a well‐characterized and commonly used probiotic.^109^ Recently, another study revealed that oral supplementation with live *LGG* can improve the antitumor response to anti‐PD‐1 in murine colon cancer and melanoma models by promoting cytotoxic CD8^+^ T‐cell activation and increasing the abundance of tumor‐infiltrating DCs.^110^ Mechanistically, *LGG* induces the production of IFN‐β through the cGAS‐STING‐TBK1‐IRF7 cascade in DCs, ultimately facilitating the antitumor activity of CD8^+^ T cells.^110^ Several studies of the commensal microbiota from people treated with PD‐1‐targeted immunotherapy showed that *Enterococcus* spp. were enriched in responding patients.^97^, ^98^ A combination of *Enterococcus faecium (E. faecium)* and anti‐PD‐L1 therapy drastically inhibited tumor growth in a B16‐F10 melanoma model.^111^ Mechanistically, peptidoglycan hydrolase secreted antigen A (SagA) from *E. faeciuma*, an NlpC/p60‐endopeptidase that can enhance the function of the intestinal barrier and pathogen tolerance, generates muropeptides to activate the NOD2 signaling pathway and modulate ICI therapy efficacy in vivo.^111^, ^112^

In addition to PD‐L1, PD‐L2 is also an important ligand in the PD‐1 signaling pathway. A recent study demonstrated that *Coprobacillus cateniformis* (*C. cateniformis*) can overcome microbial‐dependent resistance to PD‐1 pathway inhibitors and enhance the response to PD‐L1/PD‐1 immunotherapy in hormonal mice. *C. cateniformis* can down‐regulate PD‐L2 on DCs and RGMb expression on T cells, thereby relieving the PD‐L2/repulsive guidance molecule b (RGMb) pathway‐mediated suppression of T cell antitumor activity to promote antitumor immunity.^113^

## 7 THE THERAPEUTIC STRATEGY TO MODULATE GUT MICROBIOTA TO IMPROVE EFFICACY OF IMMUNOTHERAPY IN CANCER TREATMENT

With the development of multiomics sequencing and artificial intelligence, the molecular mechanisms underlying the regulation of tumor immunity and immunotherapy efficacy by the gut microbiota have been thoroughly elucidated.^114^ Therefore, manipulating microbial activity to improve the efficacy of immunotherapy has become a promising strategy. Current therapeutic strategies to improve immunotherapy by targeting the gut microbiota focus on the following areas: (1) fecal microbiota transplantation (FMT), (2) prebiotics and probiotics, (3) engineered microbiomes, (4) and other strategies, such as dietary intervention. Numerous ongoing and planned clinical trials are working to improve immunotherapy responses to cancer by directly controlling the gut microbiota of patients (Table 1).

**Table 1:** Clinical trials of gut microbiota in combination with immunotherapy in cancer treatment.

NCT number	Cancer types	Offical title	Intervention	*n*	Stage
NCT04758507	Renal cell carcinoma	Targeting gut microbiota to improve efficacy of immune checkpoint inhibitors in patients with advanced renal cell carcinoma	Biological: donor FMT Other: placebo fmt	50	Phase 1–2
NCT05690048	Hcc	Fecal microbiota transfer in liver cancer to overcome resistance to atezolizumab/bevacizumab (flora)	Drug: FMT, Vancomycin oral capsule, atezolizumab +bevacizumab, placebo vancomycin oral capsule, placebo FMT	48	Phase 2
NCT05502913	Metastatic lung cancer	Fecal microbiota transplantation to improve efficacy of immune checkpoint inhibitors in metastatic lung cancer	Drug: antibiotics Other: FMT	80	Phase 2
NCT03870607	Anal cancer squamous cell	A randomized phase ii study of the administration of prebiotics and probiotics during definitive treatment with chemotherapy‐radiotherapy for patients with squamous cell carcinoma of the anal canal (bisquit)	Dietary supplement: prebiotics in combination with probiotics	75	Phase 2
NCT05303493	Advanced NSCLC stage IV and melanoma stage IV	Phase I trial of camu camu prebiotic and immune checkpoint inhibition in patients with non‐small cell lung cancer and melanoma	Biological: camu camu capsules (camu camu powder encapsulated (500 mg each) + ICI	45	Phase 1
NCT05220124	Bladder urothelial carcinoma	An open label, randomized control study of probiotics administration in the immunotherapy of urothelial bladder carcinoma	Drug: live combined (*Bifidobacterium*, *Lactobacillus*, and *Enterococcus* capsules)	190	Phase 4
NCT05557240	Glioblastoma	Clinical study on the effect of neoantigens on the therapeutic efficacy and intestinal microbiota in patients with newly diagnosed glioma	Drug: neopepvaccine1 plus poly‐ICLC, neopepvaccine2 plus poly‐ICLC	10	Not applicable
NCT05998447	Biliary tract cancer	A phase II study to evaluate the safety and the efficacy of gen‐001 in combination with pembrolizumab for patients with advanced refractory biliary tract cancer	Drug: gen‐001, pembrolizumab, mfolfox	148	Phase 2
NCT05759741	Rectal cancer	Alterations of gut microbiome, function, and its intervention after total mesorectal excision with defunctioning ileostomy for mid‐low rectal cancer	Drug: miyairi 588	64	Early phase 1
NCT03435952	Solid tumor	A phase Ib investigation of pembrolizumab in combination with intratumoral injection of *Clostridium novyi*‐nt in patients with treatment‐refractory solid tumors	Drug: pembrolizumab, doxycycline Biological: *Clostridium noyyi*‐NT	18	Phase 1

FMT is widely used for its faster and more effective role in reconstitution of the gut microbiota and has proved successful in reversing resistance to ICI treatment.^115^, ^116^ A phase I clinical trial confirms the safety, feasibility, and potential effectiveness of FMT treatment in immunotherapy. Three of 10 metastatic melanoma patients unresponsive to PD‐1 blockade who were treated with FMT and re‐induced anti‐PD‐1 therapy showed a decrease in tumor volume and none of the 10 developed irAEs.^116^ Recently, another multicenter phase I clinical trial shows a new promise in FMT from healthy donor combined with anti‐PD‐1 for Advanced Melanoma Patients. The results showed that 13 of 20 patients (previously untreated) showed objective response (OR), including four complete response (CR).^117^ Although the outcomes of FMT‐treated patients receiving immunotherapy are encouraging, its long‐term safety and finding suitable FMT donors remain challenges for researchers.^118^, ^119^ In addition to FMT, using prebiotics or probiotics is another strategy that is being studied in the context of cancer immunotherapy. Prebiotics can act as nutrients for gut microbes to enhance the gut microbiota and thus improve the efficacy of ICI in cancer treatment.^120^, ^121^, ^122^ For example, ginseng polysaccharides (GPs), an active component of *Panax ginseng*, can improve the efficacy of αPD‐1 monoclonal antibody (mAb) in antitumor treatment through an increase in the microbial‐derived valeric acid and a decrease in l‐kynurenine, as well as the ratio of Kyn/Trp.^121^ Probiotics, such as *Bifidobacterium* and *Lactobacillus* species, are live microorganisms that can provide health benefits to the host when ingested in sufficient quantities.^123^ A recent study showed that oral administration of *Lr* combined with ICI immunotherapy significantly inhibited tumor growth in a preclinical melanoma mouse model.^95^ Engineered microbiomes, including gene‐attenuated, nutrient‐deficient and elicitable *Bifidobacteria*, *Escherichia coli*, *Salmonella*, and *Listeria* have been converted and shown antitumor effects in preclinical studies.^124^, ^125^, ^126^ Moreover, dietary intervention, cancer vaccines, and targeted antibiotic strategies can also improve the immune response to cancer as emerging therapeutic strategies.^127^

## 8 CONCLUSION AND PERSPECTIVE

In summary, the gut microbiota plays a significant role in modulating the antitumor immune response and ICI therapy effects. It is important to understand how gut microbiota regulate immune function by modulating communication pathways with the host, which is a key focus of research on gut microbiota‐mediated immunity. The intestinal flora participates in crosstalk with the host immune system and in the regulation of antitumor immunity in terms of innate and adaptive immunity. Microbiota‐derived metabolites act as adjuvants to regulate the antitumor immune response and immunotherapy efficacy. Deeper understanding of the complex interactions between microbiota, metabolites, and immunity helps us to manipulate specific immunostimulatory metabolites directly derived from gut microbiota, rather than whole or specific microbial transplants, to enhance the efficacy of ICI response. Despite growing evidence that the gut microbiota influences the efficacy of immunotherapy, the field is still in its infancy, and many of the molecular mechanisms by which the gut microbiome influences the host's response to immunotherapy remain elusive. A better understanding of the mechanisms of gut microbiome‐mediated immune regulation can help improve the accuracy of therapeutic approaches and avoid adverse outcomes.

In the future, a great deal of research is still needed to gain a deeper understanding of the mechanisms of gut microbiota‐mediated antitumor immunomodulation as well as the precise immunosuppressive or immunostimulatory strains or pathways to improve the accuracy of immunotherapeutic strategies and reduce the incidence of adverse immune events. In addition to bacteria, fungi, commensal viruses, and archaea also play an important role in anticancer immunity. Focusing on the complete analysis of the whole microbiota species and their interactions may become a future direction for preclinical or clinical studies in tumor immunotherapy. Moreover, with the development of artificial intelligence and other emerging technologies, modeling, and immunotherapy prediction through the integration of macro‐genomics, transcriptomics, metabolomics, proteomics, and other multiomics analyses will provide opportunities for the development of personalized and precise medical treatments and help to elucidate the molecular mechanisms of different therapeutic responses.

## AUTHOR CONTRIBUTIONS

Qian Yin wrote the main manuscript. Jiao‐jiao Ni designed the figure. Jie‐er Ying reviewed and edited the manuscript and figure.

## CONFLICT OF INTEREST STATEMENT

The authors declare no conflict of interest.

## Data Availability

Data sharing is not applicable to this article as no datasets were generated or analyzed during the current study.
